# Adenovirus detection in Guthrie cards from paediatric leukaemia cases and controls

**DOI:** 10.1038/sj.bjc.6604714

**Published:** 2008-10-28

**Authors:** G M Vasconcelos, M Kang, M S Pombo-de-Oliveira, J D Schiffman, F Lorey, P Buffler, J L Wiemels

**Affiliations:** 1 Laboratory for Molecular Epidemiology, Department of Epidemiology and Biostatistics, University of California, San Francisco, AC-34, 1 Irving Street, San Francisco, CA 94143-0441, USA; 2 Instituto Nacional de Câncer, Programa de Hematologia e Oncologia Pediatricás, End.:Rua André Cavalcanti, 37/5° Andar-Centro, Cep: 20231-050-, Rio de Janeiro, Brazil; 3 Department of Oncological Sciences, Huntsman Cancer Institute, University of Utah, 2000 Circle of Hope (Room 4343), Salt Lake City, UT 84112, USA; 4 Genetic Diseases Screening Program, California Department of Public Health, 850 Marina Bay Parkway, Richmond, CA 94804, USA; 5 Division of Epidemiology, School of Public Health, University of California at Berkeley, 50 University Hall, MC 7360, Berkeley, CA 94720-7360, USA

**Keywords:** adenovirus, childhood leukaemia, viral aetiology, prenatal infection, Guthrie card

## Abstract

Archived neonatal blood cards (Guthrie cards) from children who later contracted leukaemia and matched normal controls were assayed for adenovirus (AdV) *C* DNA content using two highly sensitive methods. In contrast to a previous report, AdV DNA was not detected at a higher frequency among neonates who later developed leukaemia, when compared with controls.

The causes of childhood acute lymphoblastic leukaemia (ALL), the most common type of paediatric cancer in developed countries, remain unclear. Greaves *et al* hypothesised that the malignant transformation of a normal cell to leukaemia requires two separate events (Greaves, 1999; Wiemels *et al*, 1999; Mori *et al*, 2002; Greaves *et al*, 2003). The first event would occur *in utero* producing a pre-leukaemic clone, and a secondary postnatal event is required for this clone to expand into full-blown disease. Either the pattern of normal childhood infections (the ‘delayed-infection’ hypothesis; Greaves, 1988) or specific infectious organisms (the ‘population mixing’ hypothesis; Kinlen, 1988) are most likely important modifiers of one or both of these events. Concerning the first event, one potential mechanism might involve a transforming response following maternal transmission of infection to the fetus. In this model, the infection in the fetus could induce a genomic instability favouring the onset of chromosomal aberrations or other mutations (Smith, 1997; Smith *et al*, 1997).

Molecular screening for specific candidate viruses or virus families has produced negative results (as reviewed by Greaves, 2006). Representative difference analysis (RDA) has been used to screen for anonymous, non-human sequences in the genome of common ALL (cALL) samples and no foreign sequences were detected (Mackenzie *et al*, 2006). In addition, degenerate PCR screens have not implicated a higher frequency of infection in leukaemia cells than in blood cells from children without leukaemia (MacKenzie *et al*, 2001). These data seem to provide evidence against direct viral transformation. These approaches are not infallible; however, it is possible that a very small virus or proviral component might be involved or that a transforming virus has operated through a ‘hit-and-run’ mechanism.

Human adenoviruses (AdVs) cause various common diseases in adults and children such as conjunctivitis, respiratory infections and diarrhoea (Wadell, 1984). Although AdV infections have never been convincingly linked to human oncogenesis, the transforming properties of human AdVs are well documented in animal models (Endter and Dobner, 2004). Nevels *et al* (2001) showed that AdV infections may contribute to the development of some human tumours through a mutagenesis-based hit-and-run mechanism resulting in tumours that do not carry viral genes and proteins. Other possible mechanism by which AdV could transform by a ‘hit-and-run’ mechanism is suggested by its stimulatory effects on DNA methyltransferase 1 (DNMT1) (Burgers *et al*, 2007) and the profound DNA methylation changes that occur in rodent AdV cancer models (Doerfler, 2006). Data indicating a possible role of AdVs in human oncogenesis are restricted to the investigation of serotype 5 (a *C* class virus), and little is known about the oncogenic potential of other members of the AdV family. Recently, the first evidence for the possible contribution of AdV to the multistep process of tumour pathogenesis was shown in some paediatric tumour entities but not leukaemia (Kosulin *et al*, 2007).

A provocative recent report (Gustafsson *et al*, 2007) demonstrated an increased frequency of group-C AdV in Guthrie cards from children who developed ALL. This was the first description of a very high frequency of viral DNA present in Guthrie cards, and this same group found no other viral DNAs in previous Guthrie card studies (Priftakis *et al*, 2003; Bogdanovic *et al*, 2004; Isa *et al*, 2004; Gustafsson *et al*, 2006). As AdV DNA was found only in 2 out of 130 leukaemia tumour samples in one report (Kosulin *et al*, 2007), a hit-and-run mechanism is the only likely possibility. In this study, we sought to confirm the Guthrie card screening result in a patient population in California.

## Materials and methods

### Patients

Guthrie cards from 89 children who later developed acute lymphocytic leukaemia and 100 healthy children were screened. Cards were obtained from a single centralized repository where they were stored dry at −20°C. The leukaemia cases, from which the 89 Guthrie cards were derived, were selected from a population-based case series: the Northern California Childhood leukaemia study (Chang *et al*, 2006). The leukaemia patients were between 1 month and 14 years of age at diagnosis; median of 4 years old. Controls were derived from the same population as the cases and matched by birthdates, gender, ethnicity and geographic region of birth; therefore, with the same age range and median age as the cases. Written informed consent was obtained from all case and control parents, and all studies were reviewed and approved by the appropriate institutional review boards.

### DNA extraction from Guthrie cards

A one-eighth portion of a 1.5 cm^2^ card was cut and DNA was isolated with QIAamp Micro Kit (Qiagen, Hilden, Germany) according to the manufacturer's instructions. This represents approximately 150 ng of DNA or 25 000 cells. The samples were quantified using PicoGreen DNA quantification reagent (Invitrogen, Carlsbad, CA, USA).

### Real-time quantitative PCR

Quantitative analysis of Guthrie card DNA was performed using real-time quantitative PCR for AdV *hexon* gene as described previously (Ebner *et al*, 2005) with slight modifications. Briefly, three different primer/probe systems were used to detect Adv species A, C and F in one single reaction. Polymerase chain reactions were set up in a total volume of 20 *μ*l, including 1 × TaqMan Universal Master Mix (Applied Biosystems, Foster City, CA, USA), 0.2 *μ*M probes, 0.3 or 0.9 *μ*M of primers and 20 or 50 ng of template DNA. The TaqMan probes used were labelled with 6-carboxyfluorescein at the 5′-end and with blackhole quencher at the 3′-end (MWG Biotech Inc., Huntsville, AL, USA). Amplifications were carried out using the ABI Prism 7900 HT (Applied Biosystems) for a total of 50 cycles. After an initial denaturation step for 10 min at 95°C, each cycle consisted of denaturation for 15 s at 95°C and annealing and primer extension for 60 s at 60°C. Strict precautions were undertaken to prevent contamination of PCRs with exogenous products. To reduce the risk of false-positive results due to contamination with PCR products, dTTP was partially replaced by dUTP in the reaction master mixture and a dUTP glycosylase step was performed before each PCR. Each DNA patient sample was analysed in duplicate, standard dilutions of AdV-5 were run in quadruplicates and negative controls were included in each assay. DNA derived from AdV type 5 (AdV-5) (cat. no. 7K0013, Advanced Biotechnologies Inc., Columbia, MD, USA) served as a quantitated positive control. For the quantification of virus copies, external standard curves were established using 10-fold serial dilutions of quantified virus DNA preparations corresponding to defined virus particle equivalents. As a separate internal control, we used an *Alu* element-based assay, which amplifies the *Intra-Yd6* sequence of human DNA (Hansen *et al*, 2007).

### Semi-nested PCR

The AdV *fiber* gene (a polymorphic gene that can be used to distinguish AdV species) was screened in Guthrie card samples by using semi-nested PCR. The PCR amplifications were carried out in a final volume of 50 *μ*l consisting of 1 × PCR buffer, 2.25 mM MgCl_2_, 0.2 mM each deoxynucleosidetriphosphate (Applied Biosystems), 2.5 U of *Taq Gold* polymerase (Applied Biosystems), 150 nM each primer (Garnett *et al*, 2002) and 20 ng of template DNA. Polymerase chain reaction amplification was carried out with 1 cycle at 95°C for 4 min, 35 cycles of 95°C for 45 s, 56°C for 45 s and 72°C for 45 s, followed by 1 cycle at 72°C for 3 min. Following the initial cycle of PCR, 1 *μ*l of primary PCR product was used in a second amplification. The second PCR amplification was done for 25 cycles. Polymerase chain reaction products were visualised on a 1.8% agarose gel stained with ethidium bromide. The *fiber* primary PCR product was 408 bp, and the semi-nested PCR product was 175 bp.

## Results

Eighty of the 89 cases had B-precursor ALL and a typical pattern of cytogenetic rearrangements (Table 1). Six patients had T-cell ALL, with three being indeterminate or biphenotypic (Table 1).

### Sensitivity of the assays

**Figure 1:**
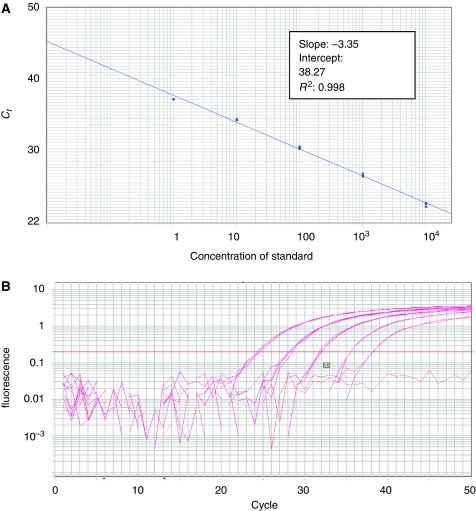
Real-time PCR standardisation of AdV ACF reaction. (**A**) Standard curve plot of the 10-fold serial dilution of AdV-5. Linearity is observed throughout the range from 1 to 10^4^ copies per reaction. (**B**) Amplification curves of the *hexon* gene with AdV-5 control DNA. The control DNA is a certified quantified standard.

**Figure 2:**
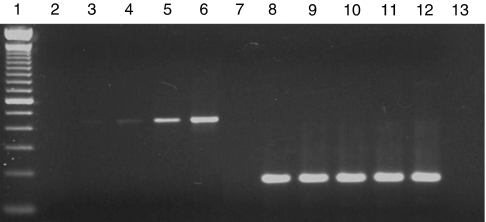
Detection of the *fiber* gene of AdV C genome by nested PCR. Serial 10-fold dilutions of the control DNA, ranging from 0.1 copy to 10^3^copies of the genome were used in the first reactions. Lane 1, molecular weight marker (100 bp); lanes 2–6, first round of amplification (0.1, 1, 10, 100 and 1000 copies, respectively); lanes 8–12, semi-nested amplification of the dilution series only; and lanes 7 and 13, negative controls.

The lower detection limit of the molecular tests described was assessed by the analysis of dilution series (ranging from 0.1 to 10^4^copies) prepared using quantified viral DNA derived from the reference strain (AdV-5). Reproducible quantification of virus copies was obtained by RQ–PCR in the presence of 1–10^4^ particles per reaction (Figure 1). When the threshold cycle values of the standard dilutions were plotted against the log_10_ of the starting copy number, the correlation coefficient values were higher than 0.990 and the slope of the line was lower than −3.34, indicating an amplification efficiency of 99–100%. With the nested PCR, the detection limit was higher. Positive results were obtained with starting quantity as low as 0.1 viral copy (Figure 2).

### RQ–PCR

DNA samples from 40 ALL patients and from 56 normal controls were analysed for the presence of AdV subgroup A, C and F DNA by real-time PCR, which is slightly less sensitive than the nested assay. The degree of homology of AdV subgroups permitted the establishment of a single RQ–PCR covering all serotypes of the species A, C and F (Ebner *et al*, 2005). Individual samples were assayed two times, on separate occasions. In the first set of reactions, 20 ng of each DNA sample (*n*=96) was run in duplicate. Then, a second set was run, with 50 ng of each DNA sample (*n*=78) also in duplicate. None of the samples contained detectable AdV DNA, and all samples amplified for *Yd6* control gene at a level consistent with the amount of DNA added (Hansen *et al*, 2007). There was no relationship between the age of the card (i.e., the age of the child) and the ease of amplification of this control gene. Owing to the negative results with this method, we moved to the two-round nested PCR approach.

### Semi-nested PCR

**Figure 3:**
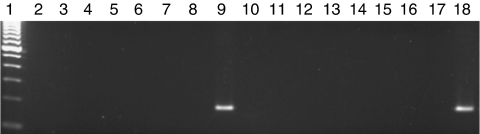
Detection of *fiber* gene of AdV-C by nested PCR in ALL samples. Results from the nested (second round) PCR are shown. Lane 1, 100 bp molecular weight marker; lanes 2–8 and 10–17, negative samples; lane 9, positive sample; and lane 18, positive control AdV-5.

Nested PCRs were carried out with DNA samples from 89 ALL patients and from 100 normal controls for detection of the *fiber* gene. The primers used in these reactions were designed to amplify all serotypes of the AdV subgroup C (Garnett *et al*, 2002). Only one of the 189 samples contained amplifiable AdV DNA (Figure 3) and it belonged to a normal control subject. It is likely that this sample carries very low AdV particle number, as it did not amplify by RQ–PCR.

## Discussion

It has been suggested that infection *in utero* with a virus that has an oncogenic potential could be involved in the initiation of ALL, by inducing genomic instability in B-lymphocytes. As AdV is a common infection in intrauterine environments (Van den Veyver *et al*, 1998; Baschat *et al*, 2003), and expression of the *E1A* and *E1B* oncogenes of AdV-5 may transform normal cells into a tumorigenic phenotype (Nevels *et al*, 2001), this virus could potentially be involved in the initial event promoting leukaemia. In this study, human AdV DNA subtypes A, C or F was not detected by PCR in the blood from Guthrie cards neither in children who had developed ALL (*n*=89) nor from healthy controls (*n*=99), except for one normal control sample. These results demonstrate a lower rate of positivity (∼0.5%) than that found for AdV in amniocentesis samples from the second trimester of normal pregnancies (∼5%; Baschat *et al*, 2003). Exposure and infection to AdV *in utero* is then likely more common than would be indicated by the current results; however, it makes sense that AdV type *C* would be less likely to be found in blood, as its natural tropism is respiratory epithelium. The virus is normally shed through stool and therefore could be deposited in amniotic fluid when a fetus is infected. All of our DNA samples contained amplifiable DNA at amounts that correlated with Pico-green assays when tested by *Alu*-element *Yd6*; therefore, PCR inhibitors do not cause our negative results from the Guthrie cards. Recently, Gustafsson *et al* (2007), using similar methodology (nested PCR and RQ–PCR) that we used in this study, showed a very high frequency of AdV group *C*-positive samples in ALL patients (27%) when compared with controls (6%). Unless the Swedish mothers are exposed to very different environmental and genetic factors that influence transmission of infection to a fetus and detection in blood, when compared with pregnant woman in California, there is no obvious explanation for this discrepancy. As sero- and molecular positivity to AdV *C* are not significantly higher in Swedish compared with USA populations (Ostlund *et al*, 2004; Arnold *et al*, 2008), we do not think that there are population differences in exposure and fetal transmission; rather the results of Gustafsson may be on tha basis of laboratory issues that are difficult to evaluate given the limited data provided (Gustafsson *et al*, 2007). The differences between the two studies may also arise from concurrent exposures that might increase infectivity of the virus in Sweden including smoking habits or respiratory diseases. Our results, presented here with positive and negative controls and a certified DNA standard, are defensible as negative, and we do not encourage further research into the detection of AdV in Guthrie cards for the purposes of studying leukaemia aetiology, at least in California.

Speculations concerning a link between infections and childhood leukaemia have been published for decades and, despite a multitude of epidemiological correlations, a definitive mechanistic explanation remains elusive. The absence of any viral DNA in Guthrie cards does not constitute negative evidence of the role of infections in leukaemia aetiology if the critical exposure is post-natal. The early postnatal period is the time period with the strongest epidemiological evidence concerning a link between infectious disease history and childhood leukaemia (Greaves, 2006). The absence of AdV DNA in Guthrie cards does not rule out a hit-and-run mechanism of this virus; its lack of detection in Guthrie cards in this study argues only against a role of this virus in prenatal infection. Future studies may examine the role of AdV and other specific viruses in the mother's and child's life. To date, however, strong evidence for specific viral involvement in childhood leukemogenesis is quite limited, compared with the large body of strong evidence linking immune modulation through unspecified common childhood infections as a protective factor.

## Figures and Tables

**Table 1:** Characteristics of leukaemia cases whose Guthrie cards were tested for the presence of adenovirus

**Patient characteristics**	***N***	**AdV DNA**
Controls	100	1
		
*Cases*	89	0
Bp ALL	80	
T ALL	6	
Unknown lineage	3	
Karyotype^a^		
Hyperdiploid (47–50)	12	
Hyperdiploid (51–67)	27	
Triploid	2	
		
*Pseudodiploid*	27	
*t*(12;21)	13	
chr11q2.3	8	
		
*Others*	4	
No abnormality	9	

AdV=adenovirus; ALL= acute lymphoblastic leukaemia; Bp ALL=B-cell precursor ALL; T ALL=T-cell ALL.

a Among Bp ALL only. Characteristics of T-cell ALL are not shown.
